# An Intramolecular Cobalt–Peptoid Complex as an Effective Catalyst for Light-Driven Water Oxidation at pH 7

**DOI:** 10.1021/acsomega.5c05267

**Published:** 2026-02-19

**Authors:** Suraj Pahar, Karunamay Majee, Galia Maayan

**Affiliations:** Schulich Faculty of Chemistry, 26747 Technion−Israel Institute of Technology, Haifa 3200008, Israel

## Abstract

Light-driven water splitting to produce hydrogen as an alternative clean renewable fuel for coping with the future energy crisis is one of the most important challenges of the 21st century. Water oxidation (WO), which is the first step in the water splitting process, is catalyzed in nature by a Mn_4_CaO_5_ cluster near pH 6. However, developing a synthetic catalyst for WO, which is based on nonprecious metal ions and can operate near neutral pH (e.g., pH 7.0), is still challenging. Herein, we demonstrate that the bioinspired intramolecular Co­(III)–peptoid complex Co**TBE** is a stable and efficient molecular catalyst for light-driven WO in phosphate buffer at pH 7 in the presence of a photosensitizer and a sacrificial electron acceptor under irradiation of blue LED light with an intensity of 1.5 mW/cm^2^, with a maximal turnover number of about 51 in 45 min. Co­(III) is coordinated and stabilized by terpyridine and bipyridine groups incorporated within the peptoid ligand, which also includes an ethanol group that acts as a proton shuttler and thus facilitates the activity at pH 7.

## Graphical abstract

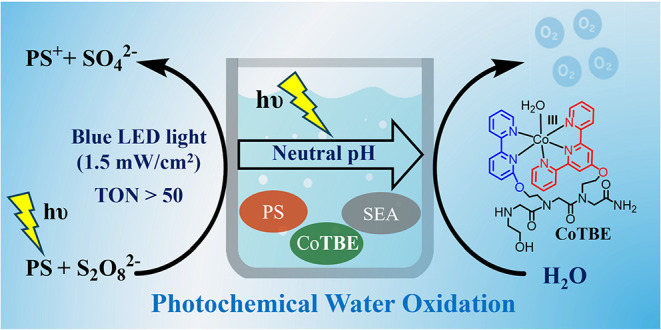

## 1 Introduction

Utilizing solar energy to split water into oxygen and hydrogen by artificial photosynthesis is the simplest way to convert solar energy into a renewable fuel source.^1^ The first step of water splitting is the four-electron oxidation of water (2H_2_O → O_2_ + 4H^+^ + 4e^–^; Δ*E*° = 1.23 V at pH 0) and the second step is proton reduction (4H^+^ + 4e^–^ → 2H_2_).^2^ Among these two steps, water oxidation (WO) is challenging both kinetically and thermodynamically, involving transfer of 4H^+^/4e^–^ and rearrangement of multiple bonds for the formation of an O–O bond.^3^ Therefore, WO is considered the bottleneck in the development of artificial photosynthetic systems for the conversion of solar energy into a chemical fuel. In recent years, significant efforts have been made to develop molecular WO catalysts. This is because molecular catalysts enable mechanistic investigations, and their stability can be enhanced by varying the coordination site abound in the metal center via ligand modifications. Although ruthenium-based molecular catalysts^4^ are still among the most studied catalysts for photochemical WO, earth-abundant first-row transition metal-based WO catalysts, such as Fe,^5^ Co,^6^ Ni,^7^ and Cu,^8^ are also being developed. However, most of the first-row transition metal complexes that have been reported as catalysts for light-driven WO operate in alkaline pH,^5,6a,6b,6d,6g,6h,8^ and there are only few reports of light-driven WO catalysts that operate at neutral conditions.^6m,7^ Among the first-row transition metal complexes, cobalt-based molecular catalysts for electro- and photochemical WO are specifically interesting because they can perform under ambient pH conditions (pH <10).^9^ However, it is a challenging task to design soluble Co-based WO catalysts due to their low stability under oxidation conditions such that they are only stable for a short period of time^10^ and some tend to get irreversibly oxidized to form Co-based oxides or hydroxides during WO, leading to heterogeneous catalytic systems.^11^ Moreover, to date, there are only few examples reporting on Co-based molecular catalysts that operate in pH = 7 toward photochemical WO, and these exhibit a low TON under high-intensity light irradiation.^6m,6n^ Therefore, developing a Co-based homogeneous light-driven WO catalyst that is stable during the light-driven WO process and is an efficient catalyst at a neutral medium (pH 7.0) is still a major challenge. To overcome this challenge, we aimed to generate a Co-based complex with a ligand environment that can stabilize the Co center and retain molecular integrity during photolysis over a long period of time in neutral pH conditions. One way to achieve this goal is to design an overall ligand environment that not only is comprised of specific ligands for Co coordination but also includes a second coordination sphere mimic around the Co center, akin to enzymes.

To mimic such ligand environments with a second coordination sphere about the metal center, our lab is using synthetic peptide mimics called peptoids,^12^ which are N-substituted glycine oligomers constructed from primary amines rather than from amino acids (Scheme 1a). Peptoids can be synthesized efficiently on a solid support using the “submonomer” method (Scheme 1b)^13^ to which a variety of side chains including the metal-binding site as well as bulky structure-directing and proton acceptor groups can be incorporated in a specific manner as a second coordination sphere mimic.^14^ Moreover, peptoids are chemically inert toward many catalytic transformations, are highly stable under various pH and oxidative conditions, and can stabilize metal ions in their high oxidation states.^15^ Hence, peptoids are excellent candidates for developing efficient bioinspired catalysts.^16^ Importantly, and specifically for WO catalysis, we found that peptoids can stabilize metal ions in their high oxidation state. Thus, we have recently reported an intermolecular Co–peptoid complex in which two peptoid molecules stabilize a Co center in the high oxidation state of 3+, enabling its activity as an efficient catalyst for electro- and photochemical WO.^6i,17a^ However, this Co­(peptoid)_2_ complex was active and stable only at pH ≥9.^17a^ Aiming to develop an efficient Co–peptoid WO catalyst that is active and stable in neutral pH conditions, we have designed the peptoid trimer **TBE** having two metal-binding ligands, namely, 2,2′;6′,2″-terpyridine (Terpy) and 2,2′-bipyridine (Bipy), for intramolecular Co binding and an ethanolic side chain, that together with the peptoid backbone, served as a second coordination sphere mimic about the Co center (Co**TBE**).^17b^ This complex acts as an efficient electrocatalyst for WO in phosphate buffer (PBS) at pH 7 with a turnover frequency (TOF) of 44 s^–1^ and a minimal overpotential of ∼430 mV and is stable over long periods of time and active for at least 10 h during electrolysis. Based on these results, we wished to explore its light-driven WO activity.

**Scheme 1:**
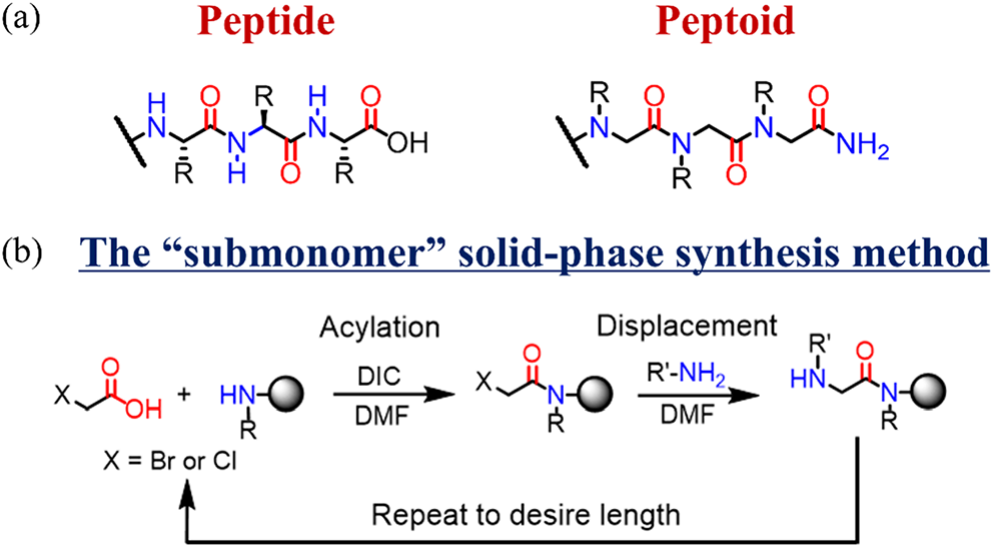
(a) Structures of the Peptide (Left) and Peptoid (Right) and (b) Solid-Phase Synthesis of the Peptoid

In this study, we report the photocatalytic activity of Co**TBE** in the presence of a Ru-based photosensitizer (PS1 or PS2, see Scheme 2) and Na_2_S_2_O_8_ as a sacrificial electron acceptor (SEA) at neutral pH (pH 7.0) conditions under irradiation of blue LED light (an intensity of 1.5 mW/cm^2^). Utilizing a blue LED light source and conducting the reaction at pH as close to natural pH as possible are desired not only toward the mimicry of the natural photosynthetic system (which utilizes sunlight and operates at pH 6) but also to enhance the overall energy efficiency of the process. However, TONs in synthetic photocatalytic systems for WO are higher at alkaline conditions due to improved deprotonation kinetics during O–O bond formation, which enhances the interaction with SEAs and increases catalyst stability and activity. Indeed, previous reports have shown that high-intensity light sources, such as xenon lamps with an intensity ranging from 300 to 500 W or light-emitting diode (LED) lamps with an intensity ≥15 mW/cm^2^ are used in the photocatalytic WO process and the reaction is mostly carried out in pH 8 or 9 (see the SI, Table S3).^6d–6h,7,11^ In contrast, our approach addresses these limitations by achieving catalytic activity using our catalyst under neutral pH conditions upon irradiation with blue LED light (an intensity of 1.5 mW/cm^2^).

**Scheme 2:**
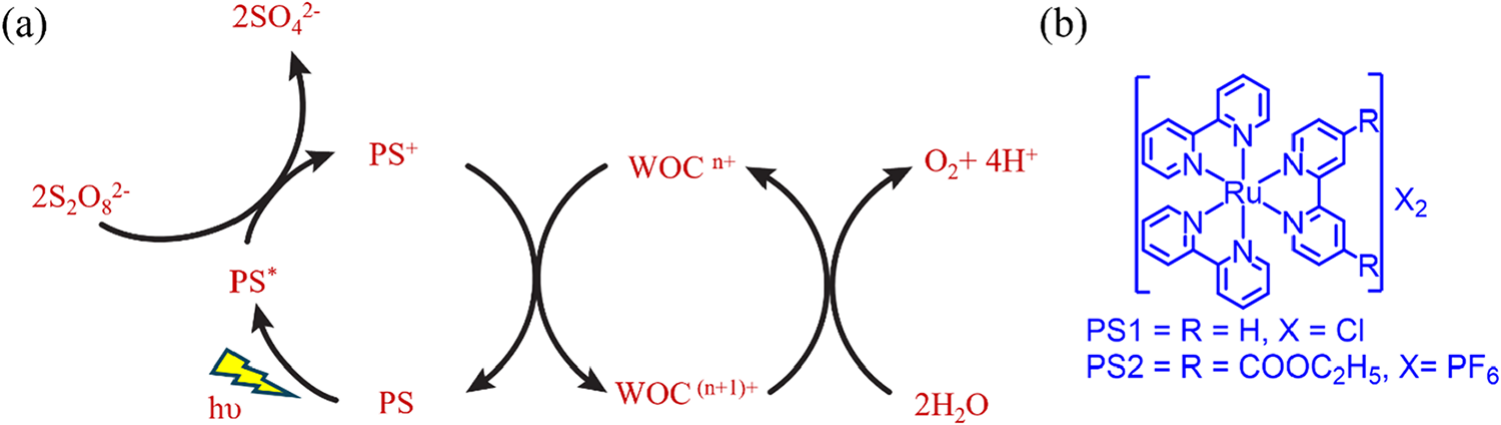
(a) General Scheme for the Photochemical Water Oxidation Reaction in a Three-Component System Containing the Water Oxidation Catalyst (WOC), Photosensitizer (PS), and Na_2_S_2_O_8_ as the Sacrificial Electron Acceptor (SEA) and (b) Structures of PS1 and PS2

The light-driven WO process is initiated with the irradiation of light where, in step 1, PS is absorbed and excited to generate PS*, which undergoes oxidative quenching by S_2_O_8_^2–^ to form PS^+^ and SO_4_^–^ radicals. Subsequently, the SO_4_^•–^ radical further oxidizes one equivalent of PS to PS^+^.^18^ Thereafter, in step 2, the successive electron transfer from the formed PS^+^ to the WO catalyst takes place to generate higher oxidation of the catalyst which is capable of splitting water molecules to evolve molecular oxygen (Scheme 2).

## 2 Methods

### 2.1 Materials and Instrumentation

Rink amide resin was acquired from Novabiochem; 6-bromo-2,2′-bipyridine and ethanolamine were obtained from Acros Organics, Israel; and bromoacetic acid and *N*,*N*′-diisopropylcarbodiimide (DIC) were sourced from Sigma–Aldrich. TFA and 4′-chloro-2,2′:6′,2″-terpyridine were acquired from Alfa Aesar. 2-(4′-Chloro-2,2′:6′,2′′-terpyridine-4′-yloxy) ethylamine and 2-(2,2′-bipyridine-6-yloxy) ethylamine were synthesized following the reported method,^17,14i^ and protection of the −OH group of ethanolamine was carried out by using a previously reported procedure.^17^ The reagents, solvents, HPLC-grade water, and acetonitrile were acquired from commercial sources and used as received without further purification, except for DMF, which was dried using molecular sieves. HPLC-grade solvents were used, and high-purity deionized water was prepared by using a nanopore Milli-Q water purification system. Aqueous phosphate buffer solutions were made by dissolving a specific portion of mono-, di-, and tribasic phosphate salts and by adjusting the pH by adding a 0.1 M NaOH solution to achieve the final pH with an ionic strength of 0.1 M. From this, phosphate buffers of lower buffer concentrations of 80, 60, 20, and 10 mM were made.

Peptoid oligomers were analyzed by reversed-phase HPLC and purified using preparative HPLC following the same conditions as previously reported.^17^ Mass spectrometry was conducted on an Advion Expression mass instrument with electrospray ionization (ESI). UV–vis measurements were performed by using an Agilent Technologies Cary 60 UV–vis spectrophotometer. An Agilent Cary 630 FTIR spectrometer was used to record the IR spectra (400–4000 cm^–1^). ^1^H NMR spectra were recorded using a Bruker NMR spectrometer AVANCE II 400. All of the conditions used for the instruments are the same as previously reported.^17^

### 2.2 Preparation and Characterization of TBE and CoTBE

Peptoid **TBE** and all other control peptoids reported in this work were synthesized manually on Rink amide resin using the submonomer approach at room temperature and were characterized by analytical HPLC and ESI-MS analysis according to the previously reported procedure.^17b^ **TBE** and all other control peptoid trimers were further purified by preparative HPLC. The corresponding cobalt complexes of the peptoid **TBE** and all other control peptoids were prepared in a methanol solution with the addition of Co­(OAc)_2_, subsequently adding NaClO_4_.^17^ Further, the prepared cobalt–peptoid complexes were characterized using the previously reported procedure.^17b^

### 2.3 Electrochemical Methods

Cyclic voltammetry (CV) experiments were performed on an EmStat PalmSens potentiostat in a three-electrode system. CV experiments were carried out using glassy carbon (GC) as the working electrode, Ag/AgCl as the reference electrode, and Pt wire as the counter electrode. All reported redox potentials in the present work have been reported versus NHE by adding 0.197 V to the obtained potential with Ag/AgCl. All CVs were performed with a scan rate of 100 mV/s except for other specifications.

### 2.4 Photochemical Methods

Photochemical water oxidation was carried out in a jacketed cylinder-shaped glass reaction vessel with a total volume of 5.39 cm^3^, which was connected to a water circulation system to maintain a consistent temperature throughout the photochemical experiment. For photochemical water oxidation experiments, initially stock solutions of all of the three components, i.e., the catalyst Co**TBE**, the sacrificial electron acceptor (SEA) Na_2_S_2_O_8_, and the photosensitizer (PS1 or PS2 as required), were made in 0.1 M aqueous phosphate buffer of pH 7 containing 20% acetonitrile. The desired concentrations of Co**TBE**, PS1 or PS2, and SEA were added to the reaction vessel (the total volume of the solution was 2.0 mL) in a phosphate buffer solution of pH 7 containing 20% acetonitrile and was sealed with a septum. The oxygen sensor needle was placed in the headspace of the photochemical cell. The reaction mixture was deaerated by continuous N_2_ gas purging inside the cell, until the sensor signal became stable. The reaction cell was then irradiated with blue LED light (1.5 mW/cm^2^) at room temperature with constant stirring. The wavelength of blue LED light used for the photochemical reaction is 470 nm, and the surface area of the irradiated spot is 8.96 cm^2^. The number of photons absorbed is 0.0528 μmol/sec within that surface area. Oxygen evolution was monitored using a FireSting GO_2_ oxygen sensor. Oxygen evolution was also measured by varying the concentration of phosphate buffer containing 20% acetonitrile (100 to 10 mM) with fixed concentrations of Co**TBE**, PS2, and SEA at pH 7, by varying the photosensitizer (0.1 to 0.8 mM) concentration with the fixed concentrations of Co**TBE** and SEA at pH 7 with an optimized buffer concentration of 20 mM, as well as by varying the catalyst (0 to 25 μM) concentration with the fixed concentration of PS2 and SEA at 20 mM phosphate buffer of pH 7 containing 20% acetonitrile. Oxygen evolution was measured by the oxygen sensor in % O_2_ and then converted into μmol using a calibration curve with the slope of 0.017659 (Figure S26).^6i^ The external quantum yield for photochemical water oxidation by the Co**TBE** catalyst during the initial 5 min was calculated by using eq 1 ^6b^

1 $$\mathrm{External Quantum Yield}\,\;=\;\frac{2\,\;\times \,\;\mathrm{Initial}\;\mathrm{rate}\,\;\mathrm{of}\,\;\mathrm{oxygen}\;\mathrm{evolution}}{\mathrm{Photon}\;\mathrm{flux}}\times \,\;100\%$$

## 3 Results and Discussion

**TBE** and its corresponding cobalt complex Co**TBE** (Figure 1) were prepared and characterized according to a previously reported procedure.^17^ The cyclic voltammetry (CV) of Co**TBE** in 0.1 M phosphate buffer (PBS) at pH 7 showed the onset potential of the catalytic wave at ∼1.23 V vs NHE, and a sharp anodic wave for catalytic WO was observed at *E* = +1.53 V vs NHE. The onset potential is very close to the Ru^III^/Ru^II^ redox potential of PS1 (*E*_1/2_ for the Ru^III^/Ru^II^ redox couple is 1.26 V vs NHE)^19^ but the catalytic WO potential is much higher than the potential of the oxidized state of PS1 (Figure S1). Therefore, photochemical WO catalyzed by Co**TBE** using PS1 is thermodynamically unfavorable in 0.1 M PBS at pH 7, indicating the need for a highly oxidizing dye for light-driven WO in this case. The Ru^III^/Ru^II^ redox potential of PS2 (E for the Ru^III^/Ru^II^ redox couple is 1.4 V vs NHE)^20^ is much higher than the onset potential of catalytic WO, suggesting that PS2 is thermodynamically capable of facilitating WO by Co**TBE**. To further confirm this hypothesis, we performed a photochemical WO experiment using PS1 and PS2 (Figure 2). The results indicated that the amount of oxygen evolved in the presence of PS1 is almost negligible and very similar to the amount of oxygen evolved in the absence of the catalyst, while the amount of oxygen evolved in the presence of PS2 is about 9 times higher in the same reaction conditions (Figure S2). Therefore, for further photocatalytic experiments, PS2 has been used as a photosensitizer.

**Figure 1:**
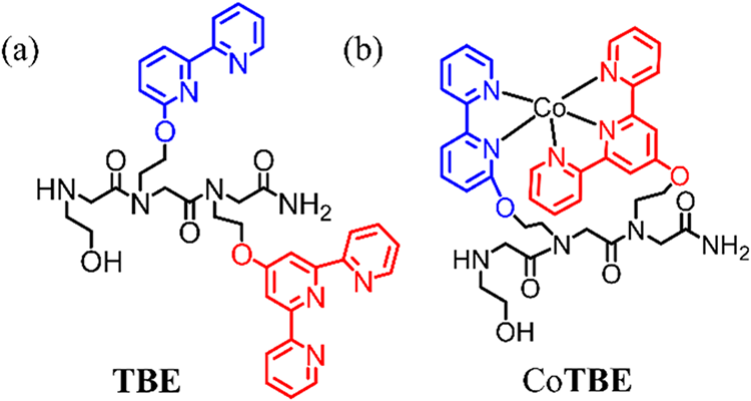
(a) Peptoid ligand **TBE** and (b) Co–peptoid complex Co**TBE**.

**Figure 2:**
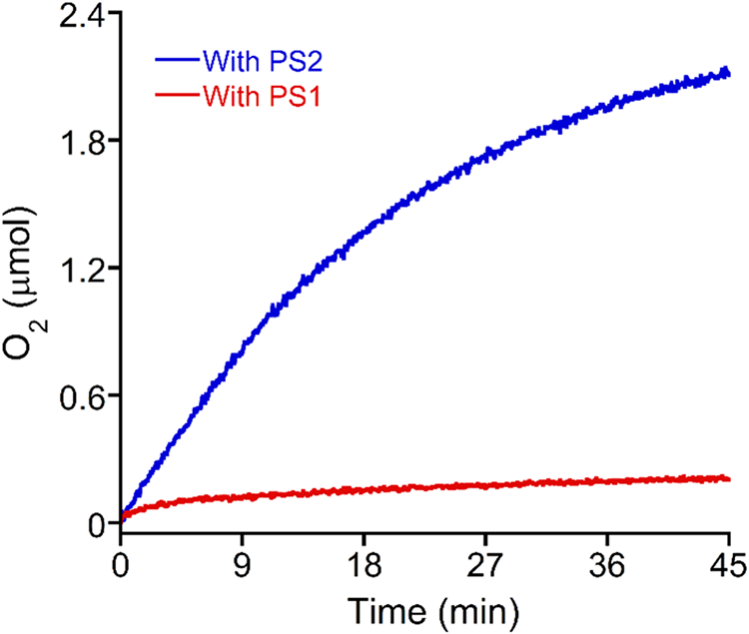
Photochemical oxygen evolution in the presence of 25 μM Co**TBE**, 0.6 mM PS1, and 3.0 mM SEA (red curve) and 25 μM Co**TBE**, 0.6 mM PS2, and 3.0 mM SEA (blue curve) under irradiation of blue light in 20 mM phosphate buffer of pH 7 containing 20% acetonitrile.

Co**TBE** can act as an efficient electrocatalyst at 0.1 M PBS at pH 7; so initially, we performed light-driven WO experiments in the same buffer condition using 1.0 mM PS2 and 5 or 4 mM SEA to obtain high TONs with these PS2:SEA concentration ratios.^21–23^ Further, we optimized the molar concentrations of PS2 and SEA to obtain significant oxygen production (Table S1). Out of all of the experiments performed, the optimal concentration was determined to be 0.6 mM PS2 and 3 mM SEA in the presence of 25 μM Co**TBE** for light-driven WO (Table S1). It was previously demonstrated that the quenching efficiency of PS2^2+*^ by SEA increases on decreasing the buffer concentration at constant SEA. This leads to the formation of more PS2^3+^ in the system, which further reacts with the catalyst to produce more oxygen and continues the overall catalytic cycle.^24^ To explore this possibility, we performed light-driven WO experiments with different phosphate buffer concentrations at constant concentrations of Co**TBE** (25 μM), PS2 (0.6 mM), and SEA (3 mM). The amount of oxygen production increased on gradually decreasing the buffer concentration from 100 to 20 mM, while a further decrease of the buffer concentration from 20 to 10 mM led to a decrease in the catalytic activity toward oxygen production (Figure S3). A reason for this might be that in concentrations lower than 20 mM, the post-photolysis pH drops below 5.0, indicating that in this buffer concentration, the pH of the system cannot be maintained and thus light-driven WO catalyzed by Co**TBE** cannot be carried out in this buffer concentration.^6i^ Therefore, further photocatalytic experiments were carried out in the optimal concentration of 20 mM phosphate buffer.

The light-driven WO activity by Co**TBE** under different concentrations of PS2 and SEA was performed. To identify the optimal concentration of PS2 required for obtaining the maximum amount of oxygen production in 20 mM phosphate buffer at pH 7, we systematically varied the concentration of PS2 from 0.1 to 0.8 mM while keeping a fixed Co**TBE** concentration of 25 μM. We found that on increasing the concentration of PS2 from 0.1 to 0.6 mM, the amount of oxygen production increased (Figures 3a and S4). This is because of the increase in the efficiency of the diffusion-controlled interaction between PS2 and Co**TBE**.^25^ We further examined the effect of the SEA concentration on the amount of oxygen formation. The amount of oxygen production increased on increasing the concentration of SEA up to 3 mM, but it decreased when the SEA concentration exceeded 3 mM (Figure S5). This is probably due to the unproductive O_2_ decomposition formed during the reaction between PS2 and SEA.^26^ Therefore, we concluded that 0.6 mM concentration of PS2 and 3 mM concentration of SEA are the optimal concentrations to obtain the highest amount of oxygen production.

**Figure 3:**
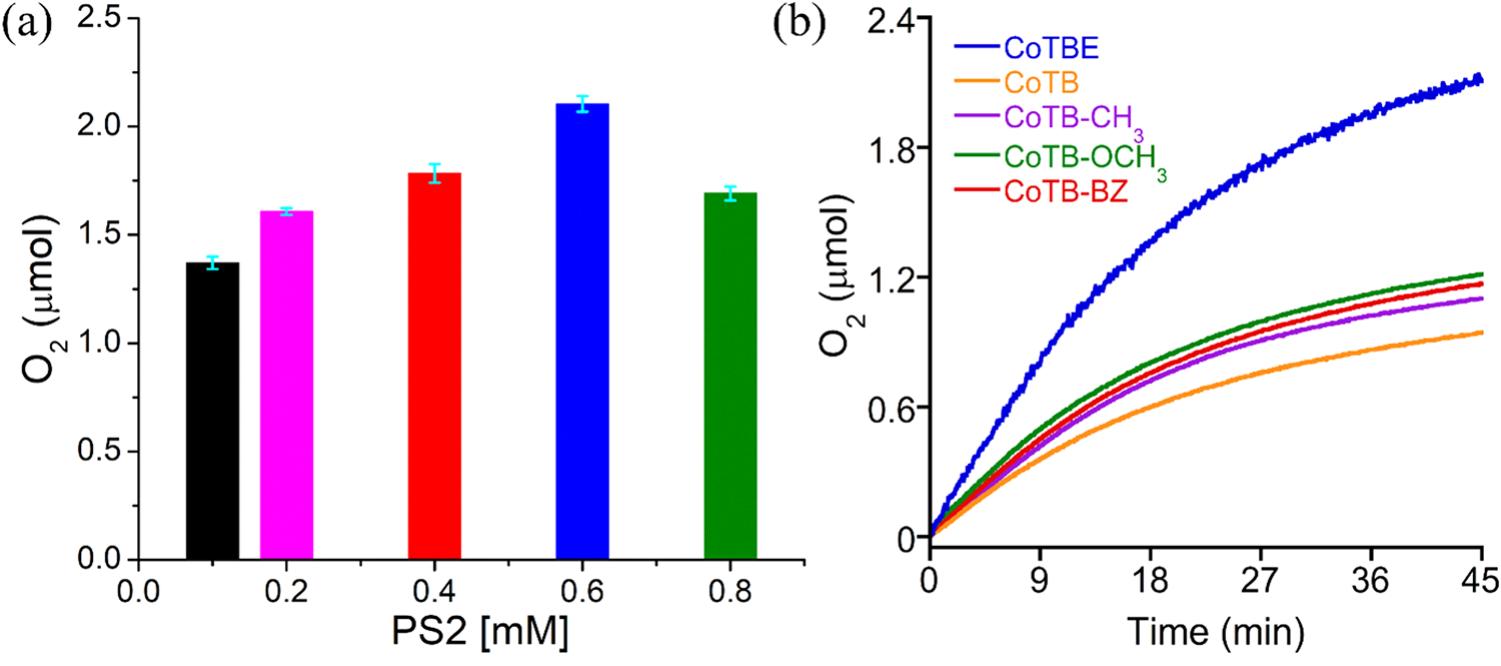
Amount of oxygen produced under irradiation of blue LED light using 25 μM Co**TBE** and 3.0 mM SEA in 20 mM phosphate buffer at pH 7 containing 20% acetonitrile and (a) various PS2 concentrations (0.1 to 0.8 mM) (b) in the presence of 0.6 mM PS2 and 25 μM Co**TB**, Co**TB-CH**_**3**_, Co**TB-OCH**_**3**_, and Co**TB-BZ**.

To explore the potential role of the ethanolic side chain as a proton shuttler that facilitates the WO process, we tested a set of control Co–peptoid complexes in which the sequence of **TBE** was modified, such that the ethanolic side chain was replaced by other groups. Thus, the activity of four additional Co-based peptoid complexes having methoxy group (Co**TB-OCH**_**3**_), methyl group (Co**TB-CH**_**3**_), or benzyl group (Co**TB-BZ**) side chains instead of the ethanol side chain, as well as Co**TB** having no additional side chain at the N-terminal, toward light-driven photochemical WO was examined in the same optimized reaction conditions used with Co**TBE**. The results indicated that when using these four control peptoid complexes, the amount of oxygen production after irradiation of light for 45 min was much lower than the amount of oxygen evolved in the presence of Co**TBE** under the same conditions (Figure 3b). Hence, we can conclude that the presence of an ethanolic side chain within the peptoid sequence plays a key role in facilitating the light-driven WO process. Furthermore, to understand the proton-relaying functionality of Co**TBE**, we have compared the oxygen evolution reaction with identical optimized conditions in a buffered medium with H_2_O or D_2_O as a solvent. We observed that in D_2_O, oxygen evolution was very slow, much slower than in H_2_O; after 45 min of reaction, the amount of oxygen evolved in D_2_O was about 30% of that evolved in H_2_O (Figure S6).^4d^ These results imply that protons play an important role in this reaction. Taken together with the results that indicate the important role of the ethanol side chain in the reaction, we can suggest that the −OH group near the catalytic center acts as a proton relay in the reaction, thus facilitating the nucleophilic attack of water to generate oxygen.

The light-driven WO experiments were further performed in different concentrations of Co**TBE** with fixed concentrations of PS2 (0.6 mM) and SEA (3 mM) in 20 mM phosphate buffer at pH 7 (Figure S7). The effect of the varying Co**TBE** concentration on the amount of oxygen evolved after 45 min of light irradiation is shown in Figure 4a. On increasing the catalyst concentration from 5 to 25 μM, the amount of evolved oxygen increased from about 0.64 to about 2.10 μmol (see also Table S2). The maximal TON, calculated by dividing the amount of evolved oxygen by the amount of catalyst used, was nearly 51 at 5 μM catalyst concentration. Table S3 shows that the majority of the reported Co-based catalysts for light-driven WO exhibit a TON of about 50, similar to that obtained with Co**TBE**, or lower than 50. However, these TONs are typically obtained under alkaline conditions (pH 8–9) and/or by using a high-intensity light source. In contrast, Co**TBE** operates in neutral pH (7.0) with blue LED light (an intensity of 1.5 mW/cm^2^). Although a few studies showed significantly higher TONs in most of them, a high-intensity Xe lamp was used as the light source, and only in one study, in which a LED light source was used, the reaction was carried out at pH 9. Thus, Co**TBE** represents itself as a unique catalyst for light-driven WO that operates efficiently in pH 7 (not >7) and under irradiation of a blue LED light source (an intensity of 1.5 mW/cm^2^), achieving a TON typically observed under higher pH and a high-intensity light source. The TOF (TOF_initial_) calculated from the TON in the first 5 min of the reaction was found to be the highest in the presence of 5 μM catalyst concentration and was determined to be 0.038 s^–1^.

**Figure 4:**
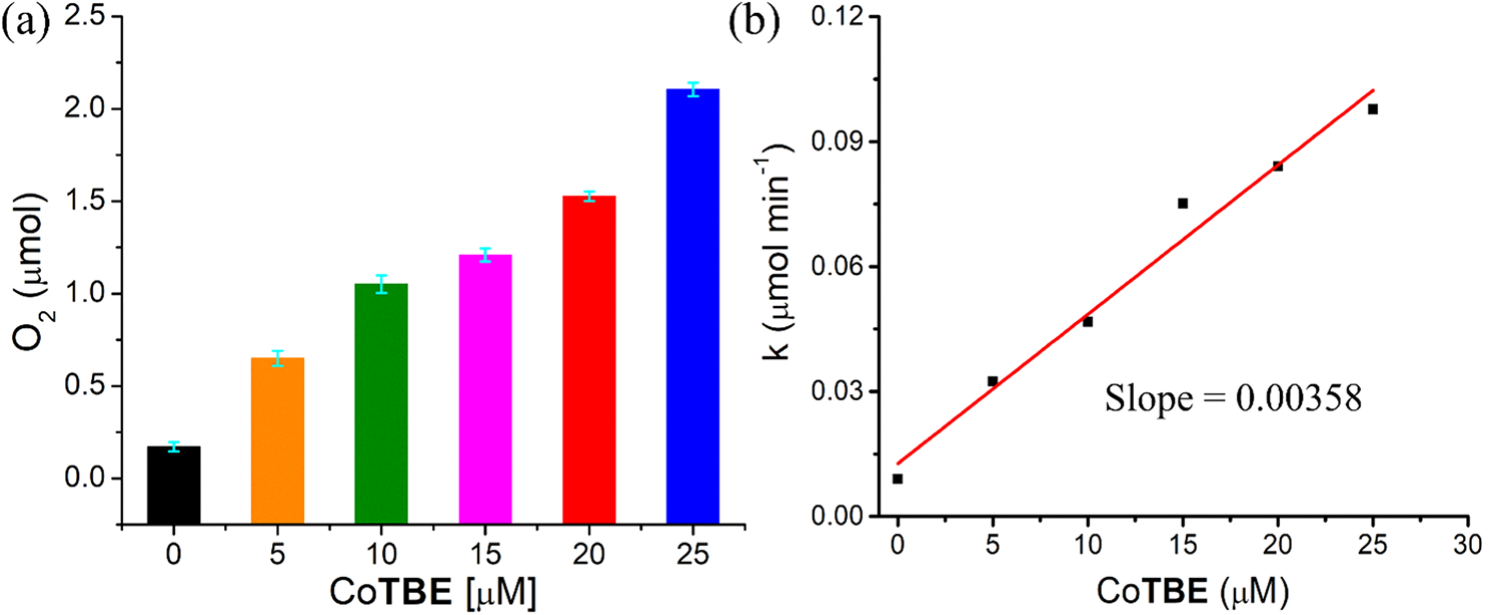
(a) Amount of oxygen produced after 45 min of light irradiation of solutions containing various concentrations of Co**TBE** (0 to 25 μM) and fixed concentrations of PS2 (0.6 mM) and SEA (3.0 mM) in 20 mM phosphate buffer at pH 7 containing 20% acetonitrile. (b) Initial rate of O_2_ formation after 5 min of light irradiation of solutions containing 0 to 25 μM Co**TBE**, 0.6 mM PS2, and 3.0 mM SEA in 20 mM phosphate buffer at pH 7 containing 20% acetonitrile.

To determine the initial rate of oxygen evolution, we looked at the initial 5 min of the light-driven WO experiments at each catalyst concentration and fitted the plot in each case to obtain a linear slope. From this linear slope, we deduced the rate of oxygen evolution in each catalyst concentration (Figures S8–S13). The obtained values were plotted against the Co**TBE** concentration and fitted again. The linear dependence of the oxygen evolution rate with the Co**TBE** concentration (Figure 4b) can suggest a first-order reaction kinetics, which corresponds to a nucleophilic attack (WNA) mechanism,^6a^ and a unimolecular reaction as the rate-determining step toward photochemical WO in these conditions.^10a^ The calculated rate of oxygen evolution was 0.00358 μmol/min, which equals 0.0597 × 10^–3^ μmol/sec. This was used to calculate the external quantum yield of the photochemical WO reaction catalyzed by Co**TBE**, using eq 1, which was found to be 0.23%.

Control experiments were conducted to confirm that all of the three components (PS2, SEA, and Co**TBE**) are necessary for efficient light-driven WO. These control experiments suggested that the absence of any of the components in the system hampered the production of oxygen, indicating that each of the three components is essential for efficient photocatalytic WO (Figures S14–S16). To prove that the reaction is initiated by exposing the reaction mixture to light, we performed an experiment with all of the three components in the presence and in the absence of light (Figure S17) and observed no oxygen evolution in the absence of light. This indicates that the WO reaction is initiated by light. Moreover, the effect of the light-driven WO reaction was further explored by turning the light source on and off in 5 min time intervals (Figure S18). During the light irradiation, the reaction between PS2 and SEA begins, leading to the production of oxidized PS2. This oxidized PS2 then further oxidized Co**TBE** to evolve oxygen. In contrast, in the absence of light, the reaction between PS2 and SEA was not initiated, further supporting the conclusion that the WO reaction starts only in the presence of light. The slight increase in oxygen evolution during the switching off period might be due to the diffusion of oxygen residue, produced during the light-on period, from the liquid phase to the headspace of the reaction vessel that was detected by the probe during the light-off period.^6i^

To explore the stability of the different components in the catalytic system, we performed UV–vis measurements of solutions containing either a mixture of Co**TBE**, PS2, and SEA or a mixture of only PS2 and SEA before and after 20 min of light irradiation, and the obtained spectra were compared (Figure S19). The observed change at 488 nm after light irradiation in both spectra indicated the decomposition of PS2 in both solutions. However, the decomposition of PS2 was much higher in the absence of Co**TBE** than in the presence of Co**TBE** (31.65 vs 7.3% decrease in its concentration, respectively). This difference is due to the effective reduction of PS2^3+^ to PS2^2+^ in the presence of Co**TBE**, which further continues the photocatalytic WO cycle.^24^

Recently, Bonnet et al. demonstrated that under light irradiation and in the presence of SEA, the photosensitizer irreversibly decomposes, forming acetic and formic acids through oxidation of its bipyridine ligands within the photosensitizer.^7^ To investigate this possibility in our system, ^1^H NMR measurements of the overall system (containing 25 μM Co**TBE**, 0.6 mM PS2, and 3.0 mM SEA in 20 mM PBS at pH 7) were carried out before and after 45 min of light irradiation, followed by lyophilization and redissolution in D_2_O (Figure S20). No characteristic peak corresponding to acetic or formic acid was detected before or after photolysis, ruling out the possible decomposition of a photosensitizer in this manner.

To further evaluate the stability of Co**TBE** under catalytic conditions, we performed a recycling experiment. Following the initial 45 min photolysis, we adjusted the pH to 7 and added fresh PS2 (0.6 mM) and SEA (3.0 mM) to the same reaction mixture and ran it for an additional 45 min under the same intensity of light irradiation as in the first reaction (Figure S21). The amount of oxygen evolved at the end of the second reaction was only 27% of the amount of oxygen evolved in the initial run with fresh Co**TBE**. Notably, our previous electrocatalytic water oxidation with Co**TBE** demonstrated that the majority of the Co**TBE** complex irreversibly transformed into inactive species during electrocatalytic water oxidation.^17b^ Taken together, these results suggest that the reduced photocatalytic activity of the system is due to the deactivation of the catalyst via the formation of inactive species. However, a minor fraction of Co**TBE** remains intact, producing a limited amount of oxygen during recycling experiments. Importantly, this result also rules out the formation of active heterogeneous species (e.g., CoO_*x*_), as such species would likely have maintained similar oxygen production during the recycling experiment.

To further support the formation of inactive species via structural changes within the catalyst that occur during the reaction, FTIR spectroscopy of the dried reaction mixture containing 25 μM Co**TBE**, 0.6 mM PS2, and 3 mM SEA before and after the photocatalytic experiment was performed (Figures S22 and S23). The spectrum shows a broad O–H stretching frequency before photolysis, indicating the presence of an ethanolic O–H group in the peptoid sequence. After photolysis, however, an additional broadening of the O–H stretching frequency was observed in the region between 3000 and 3400 cm^–1^. The further broadening might be attributed to the stretching of the O–H bond of water molecules, indicating that a water molecule coordinates to Co during photolysis. In addition, significant changes in C–N (∼1100 to 1400 cm^–1^) and C=N (∼1500 to 1700 cm ^–1^) bond stretching regions were observed in postphotolysis FTIR spectra (Figures S22 and S23), associated with structural changes in Co**TBE** during photocatalytic water oxidation. This result is very similar to our previous observation during the electrocatalytic WO with Co**TBE**, which suggested the dissociation of the Bipy center from Co during the light-driven WO reaction.^17b^

Finally, to explore the molecular integrity of Co**TBE**, we conducted dynamic light scattering (DLS) measurements of the solution before and after photolysis, in both the presence and absence of Co**TBE**. The obtained spectra were found to be identical, ruling out the possible formation of insoluble nanoparticles in the presence of Co**TBE** during photocatalytic water oxidation (Figures S24 and S25). These findings demonstrate that Co**TBE** maintains its molecular integrity under photocatalytic conditions and further support the homogeneity of the catalytic process.

## 4 Conclusions

In summary, we present here a Co-based metallopeptoid that can act as an efficient molecular light-driven WO catalyst in the presence of a photosensitizer and a sacrificial electron acceptor at neutral pH. Importantly, Co**TBE** is capable of catalyzing photochemical WO under irradiation of blue LED light (an intensity of 1.5 mW/cm^2^). Only a few examples reported the utilization of a LED light source; in two of these cases, the light intensity was not mentioned but the TON was similar to the one we obtained (about 50) albeit the reaction was carried out at higher pH (8–9) (see the SI, Table S3). In one of these cases, in which a LED light source was used but the reaction was carried out at pH 9, the authors could achieve a high TON of about 1600. In all of the other examples, mostly Xe or Hg lamps at high-intensity light of ≥300W were used (at pH 7–9).

Moreover, Co**TBE** acts as an efficient catalyst for photochemical WO under environmentally benign conditions (pH 7.0) to mimic the natural photosystem. Co**TBE** performs with a TON of 51, which is notable considering that it operates under neutral pH conditions using a blue LED light source (an intensity of 1.5 mW/cm^2^). Achieving photocatalytic activity toward WO in neutral pH and with a blue LED light source with an intensity of 1.5 mW/cm^2^ simultaneously is the major advantage of our system over other reported Co-based homogeneous catalysts for light-driven WO. Moreover, we showed that Co**TBE** is very stable during the photocatalytic process (not typical for Co-based complexes) and that the reaction is truly homogeneous. We also demonstrated that the ethanolic side chain within the peptoid sequence, which mimics an enzymatic second coordination sphere, plays a significant role in the much higher activity of Co**TBE** compared to the other peptoid complexes lacking the ethanolic side chain. Thus, our study provides valuable insight toward developing efficient biomimetic Co–peptoid complexes for homogeneous photocatalytic WO that is active in neutral pH toward renewable energy production.

## Supplementary Material



## Notes

All experimental data, including cyclic voltammetry, oxygen evolution under various conditions, control experiments, spectroscopic studies before and after photolysis, and calibration details (PDF)

## Notes

Author Contributions

## †

School of Basic Sciences, Galgotias University, Uttar Pradesh 203201, India.
