# Supplementary material for: An Intramolecular Cobalt–Peptoid Complex as an Effective Catalyst for Light-Driven Water Oxidation at pH 7
Source: ACS Omega. 2026 Feb 19;11(8):12992–9. doi: 10.1021/acsomega.5c05267 (PMC12961500; doi:10.1021/acsomega.5c05267)
Supplement: Supplementary file 1 [file ao5c05267_si_001.pdf]

Supporting Information

An Intramolecular Cobalt-Peptoid Complex as an Effective Catalyst for Light-Driven Water Oxidation at pH 7

Suraj Pahar, [a] Karunamay Majee† [a] and Galia Maayan*[a]

a: Schulich Faculty of Chemistry, Technion–Israel Institute of Technology, Haifa, 3200008, Israel.

† Current affiliation: School of Basic Sciences, Galgotias University, Uttar Pradesh, 203201, India.

*: corresponding author and email: gm92@technion.ac.il

Table of Contents

**Figure S1.**	Cyclic Voltammetry (CV) of Co**TBE,** PS1 and PS2
**Figure S2.**	Evolution of O_2 with PS1 and PS2
**Figure S3.**	Oxygen evolution in different phosphate buffer concentration
**Figure S4.**	Variation of PS2 concentration
**Figure S5.**	Variation of SEA concentration
**Figure S6.**	Oxygen evolution in buffered medium with H_2O and D_2O as solvent
**Figure S7.**	Oxygen production for different Co**TBE** concentration
**Figure S8-13.**	Linear fitting for the initial 5 min irradiation with different Co**TBE** concentration
**Figure S14.**	Photochemical oxygen evolution in presence and in absence of Co**TBE**
**Figure S15.**	Photochemical oxygen evolution in presence and in absence of PS2
**Figure S16.**	Photochemical oxygen evolution in presence and in absence of SEA
**Figure S17.**	Photochemical oxygen evolution with and without irradiation of blue LED light
**Figure S18.**	Light controlled photochemical water oxidation experiment
**Figure S19.**	UV-Vis spectra before and after photolysis
**Figure S20.**	^{1}H NMR of before and after photolysis
**Figure S21.**	Recycling experiment
**Figure S22-23.**	FTIR spectra of before and after photolysis
**Figure S24-25.**	DLS spectra of before and after photolysis, with and without Co**TBE**
**Figure S26.**	Calibration curve for the measurement of evolved O_2 in µL from %O_2

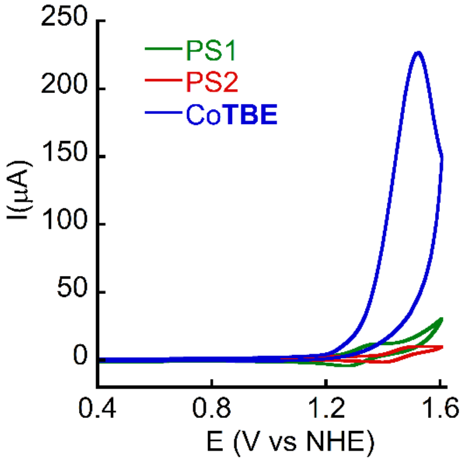

Figure S1: CVs of 0.5 mM Co**TBE,** 0.5 mM PS1 and 0.5 mM PS2 in 0.1 M PBS at pH 7.0.

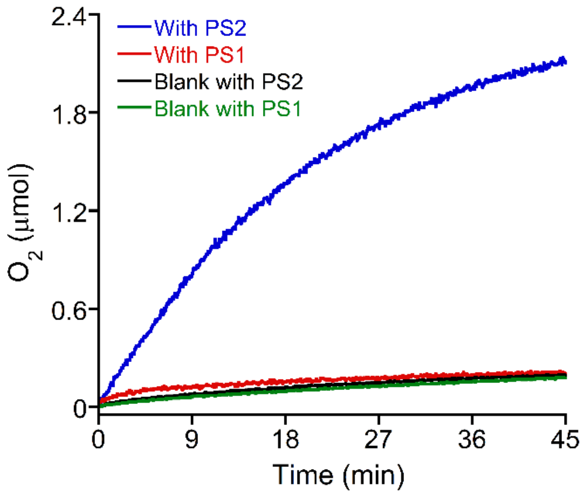

Figure S2: Photochemical oxygen evolution in the presence of 25 μM Co**TBE**, 0.6 mM PS1 and 3.0 mM SEA (red curve), 0.6 mM PS1 and 3.0 mM SEA (green curve), 25 μM Co**TBE**, 0.6 mM PS2 and 3.0 mM SEA (blue curve) and 0.6 mM PS2 and 3.0 mM SEA (black curve) under of blue-light irradiation in 20 mM phosphate buffer of pH 7 containing 20% acetonitrile.

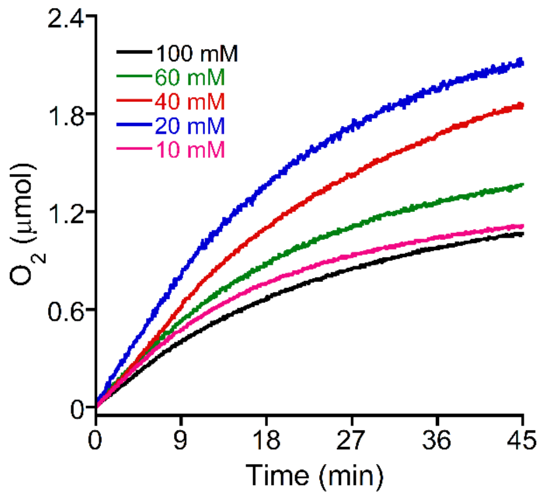

Figure S3. Evolution of O_2 at different phosphate buffer concentrations (100 mM, 60 mM, 40 mM, 20 mM and 10 mM) of pH 7.0 containing 20% acetonitrile in the presence of 25 µM Co**TBE**, 0.6 mM PS2 and 3.0 mM SEA under the irradiation of blue LED light.

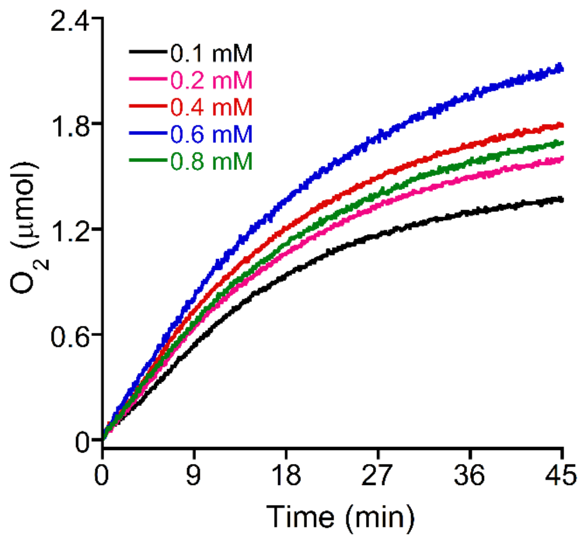

Figure S4. Variation of PS2 concentration (0.1 mM to 0.8 mM) for oxygen evolution with a fixed concentration Co**TBE** (25 µM) and SEA (3.0 mM) under the irradiation of blue LED light in 20 mM phosphate buffer of pH 7 containing 20% acetonitrile.

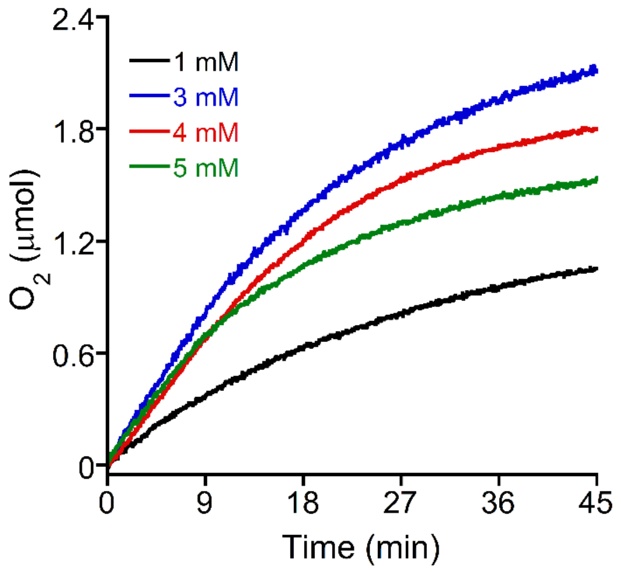

Figure S5. Variation of SEA concentration (1 mM to 5 mM) for oxygen evolution with a fixed concentration Co**TBE** (25 µM) and PS2 (0.6 mM) under the irradiation of blue LED light in 20 mM phosphate buffer of pH 7 containing 20% acetonitrile.

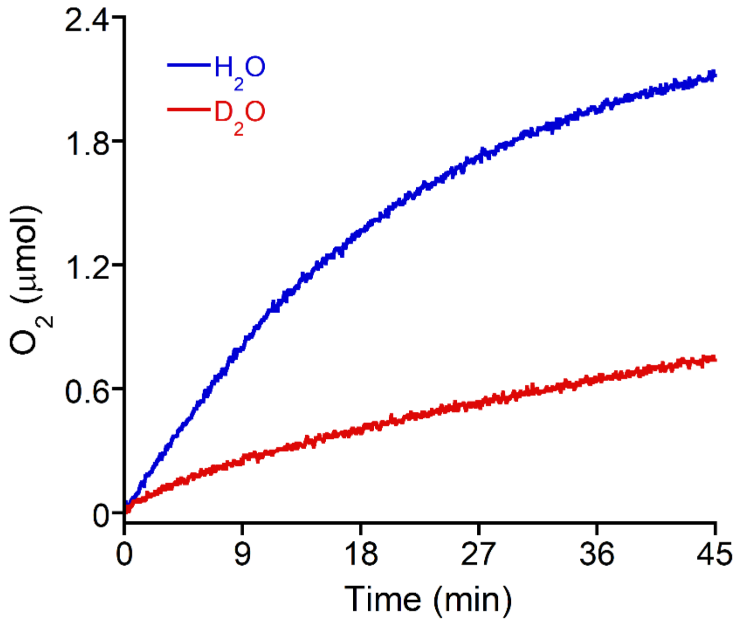

Figure S6. Photochemical oxygen evolution in the presence of 25 µM Co**TBE**, 0.6 mM PS2 and 3.0 mM SEA in buffered medium with H_2O (blue curve) and D_2O as the solvent (Red curve) under the irradiation of blue light in 20 mM phosphate buffer of pH 7 containing 20% acetonitrile.

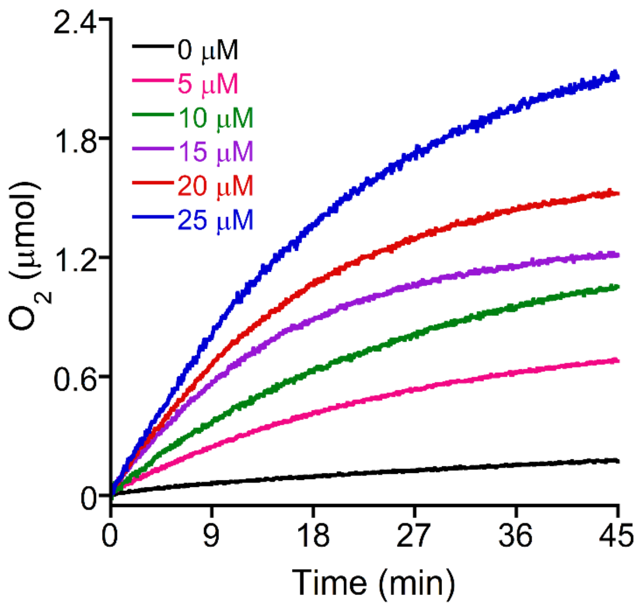

Figure S7. Oxygen production for different catalyst concentration (0 μM to 25 μM) with respect to the blank (in the absence of catalyst) to determine TONs for a fixed concentration of PS2 (0.6 mM) and SEA (3.0 mM) $Na_2S_2O_8$ under the irradiation of blue LED light in 20 mM phosphate buffer of pH 7 containing 20% acetonitrile.

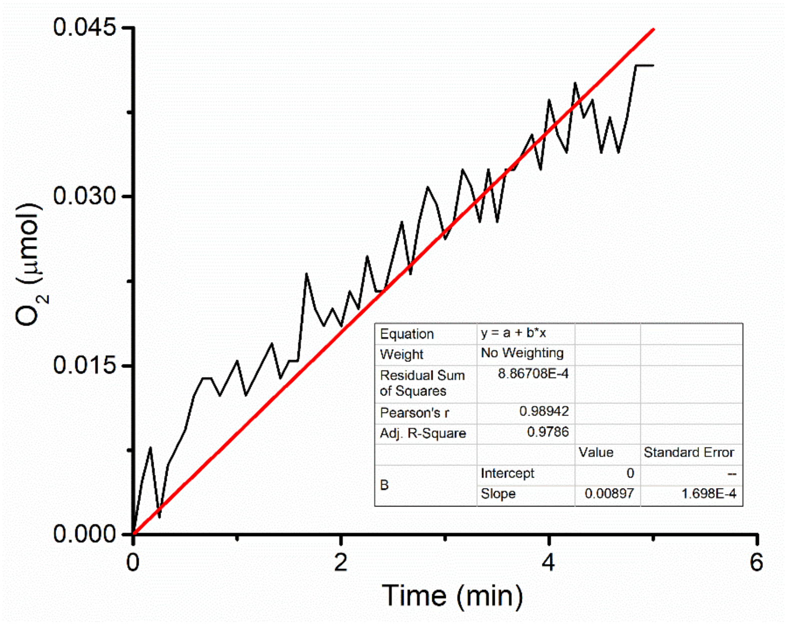

Figure S8. Linear fitting (red line) for the initial 5 min irradiation of 0 μM Co**TBE** i.e., in absence of catalyst (black line) for a fixed concentration of PS2 (0.6 mM) and SEA (3.0 mM) under the irradiation of blue LED light in 20 mM phosphate buffer of pH 7 containing 20% acetonitrile.

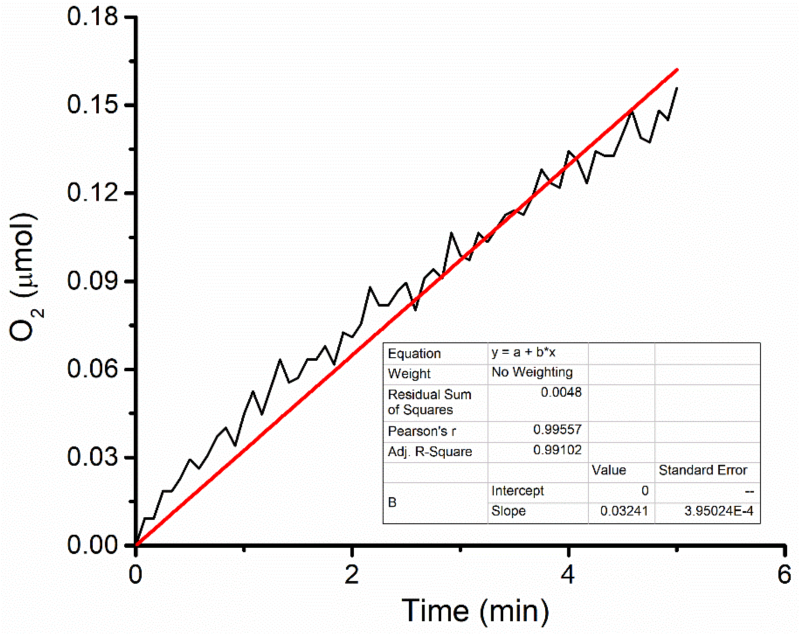

Figure S9. Linear fitting (red line) for the initial 5 min irradiation of 5 µM Co**TBE** (black line) for a fixed concentration of PS2 (0.6 mM) and SEA (3.0 mM) under the irradiation of blue LED light in 20 mM phosphate buffer of pH 7 containing 20% acetonitrile.

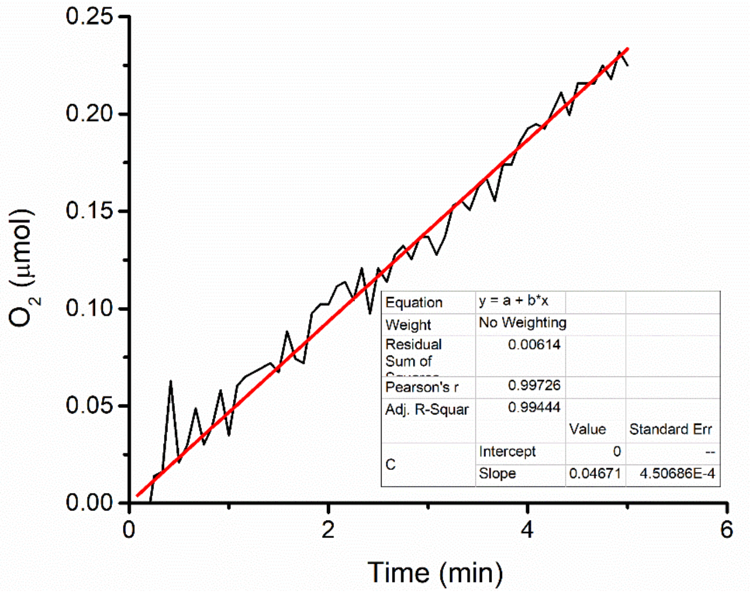

Figure S10. Linear fitting (red line) for the initial 5 min irradiation of 10 µM Co**TBE** (black line) for a fixed concentration of PS2 (0.6 mM) and SEA (3.0 mM) under the irradiation of blue LED light in 20 mM phosphate buffer of pH 7 containing 20% acetonitrile.

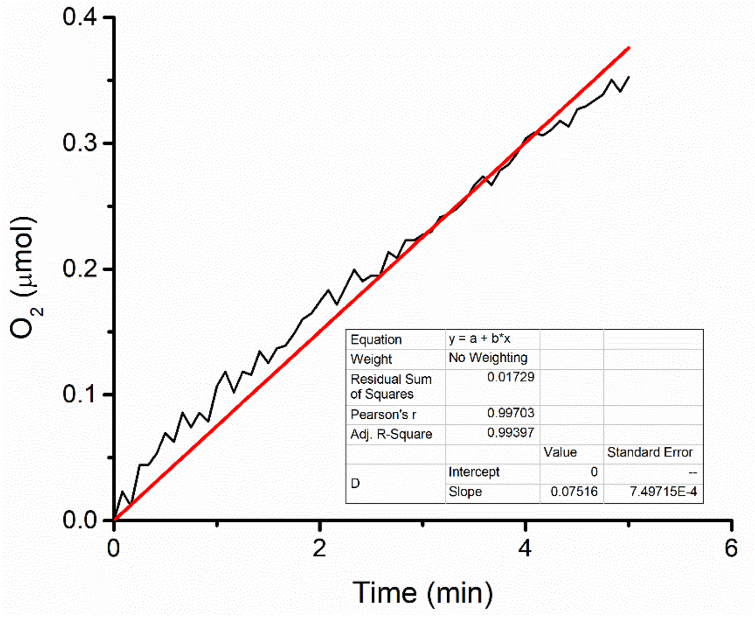

Figure S11. Linear fitting (red line) for the initial 5 min irradiation of 15 µM Co**TBE** (black line) for a fixed concentration of PS2 (0.6 mM) and SEA (3.0 mM) under the irradiation of blue LED light in 20 mM phosphate buffer of pH 7 containing 20% acetonitrile.

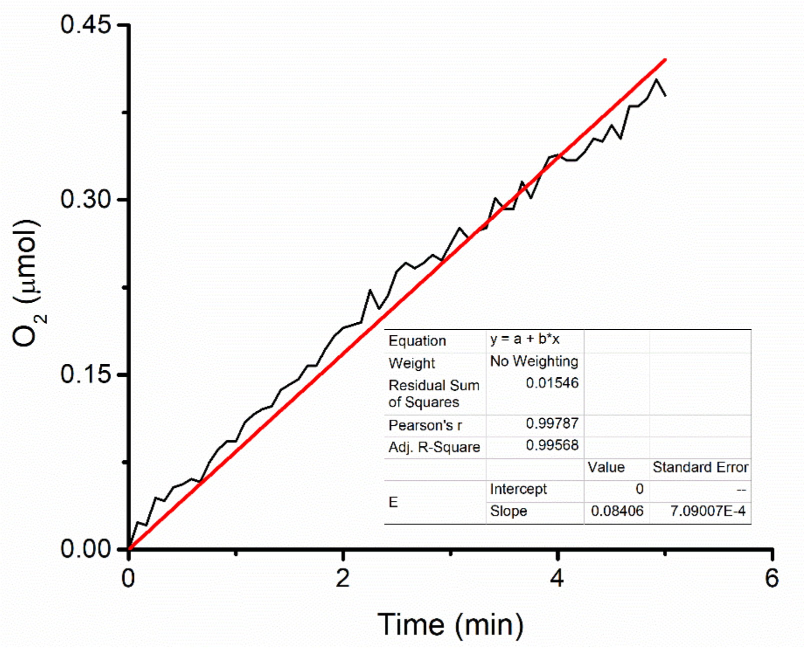

Figure S12. Linear fitting (red line) for the initial 5 min irradiation of 20 µM Co**TBE** (black line) for a fixed concentration of PS2 (0.6 mM) and SEA (3.0 mM) under the irradiation of blue LED light in 20 mM phosphate buffer of pH 7 containing 20% acetonitrile.

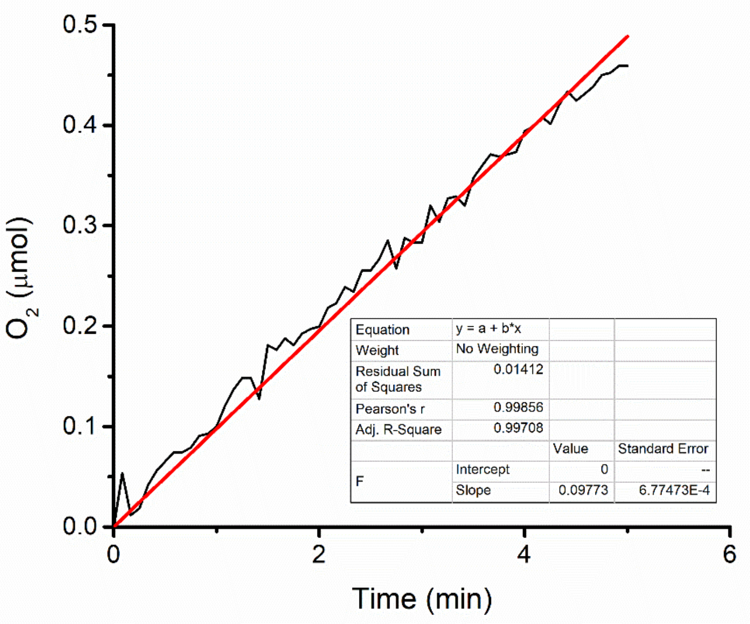

Figure S13. Linear fitting (red line) for the initial 5 min irradiation of 25 µM Co**TBE** (black line) for a fixed concentration of PS2 (0.6 mM) and SEA (3.0 mM) under the irradiation of blue LED light in 20 mM phosphate buffer of pH 7 containing 20% acetonitrile.

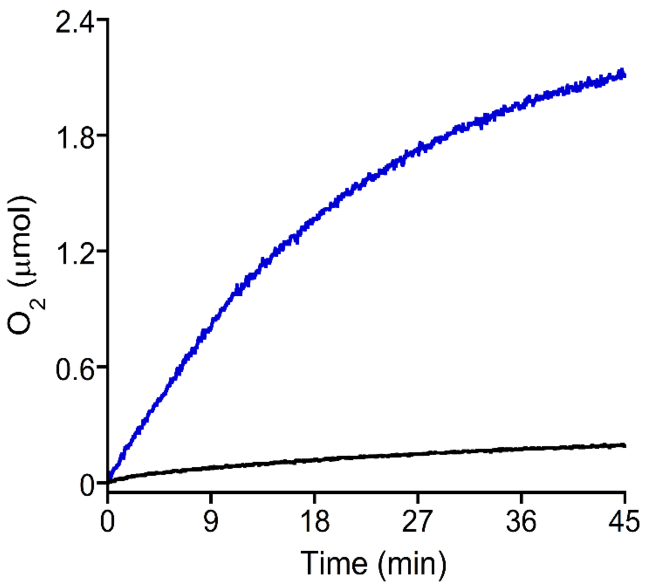

Figure S14. Photochemical oxygen evolution in the presence of 25 µM Co**TBE**, 0.6 mM PS2 and 3.0 mM SEA (blue curve) as compared to 0.6 mM PS2 and 3.0 mM $Na_2S_2O_8$ (black curve) under the irradiation of blue light in 20 mM phosphate buffer of pH 7 containing 20% acetonitrile.

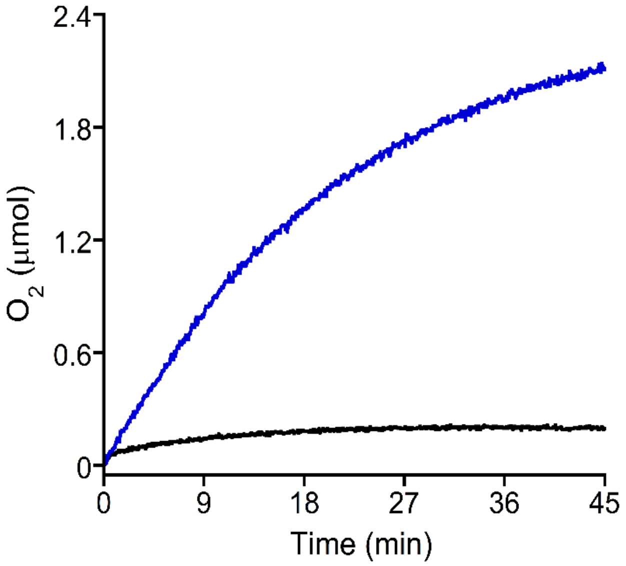

Figure S15. Photochemical oxygen evolution in the presence of 25 µM Co**TBE**, 0.6 mM PS2 and 3.0 mM SEA (blue curve) as compared to 25 µM Co**TBE** and 3.0 mM $Na_2S_2O_8$ (black curve) under the irradiation of blue light in 20 mM phosphate buffer of pH 7 containing 20% acetonitrile.

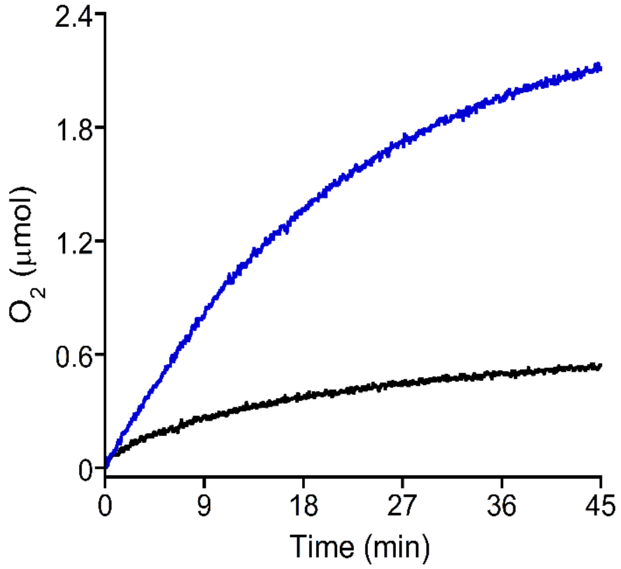

Figure S16. Photochemical oxygen evolution in the presence of 25 µM Co**TBE**, 0.6 mM PS2 and 3.0 mM SEA (blue curve) as compared to 25 µM Co**TBE** and 0.6 mM PS2 (black curve) under the irradiation of blue light in 20 mM phosphate buffer of pH 7 containing 20% acetonitrile.

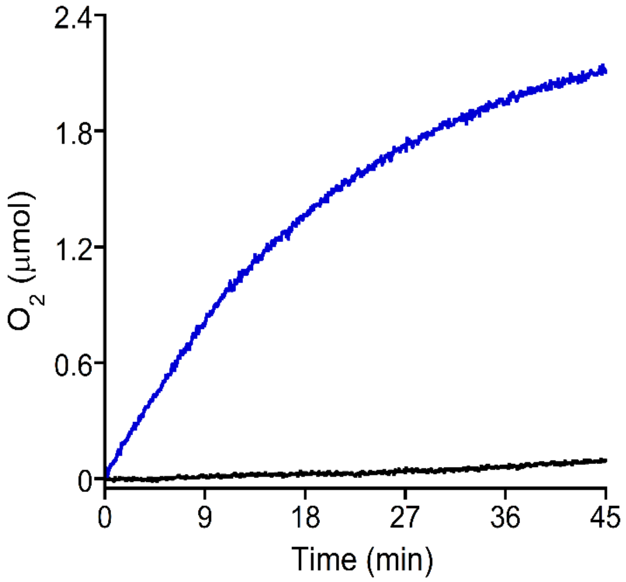

Figure S17. Photochemical oxygen evolution in the presence of 25 µM Co**TBE**, 0.6 mM PS2 and 3.0 mM SEA in 20 mM phosphate buffer of pH 7 containing 20% acetonitrile with blue-light irradiation (blue curve) and without irradiation of blue LED light (black curve).

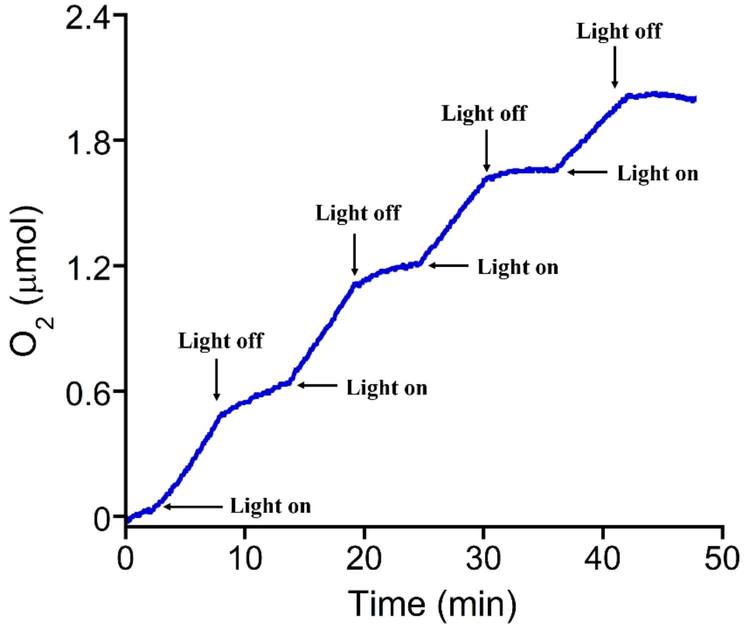

Figure S18. Light controlled photochemical water oxidation experiment in the presence of 25 µM Co**TBE**, 0.6 mM PS2 and 3.0 mM SEA in 20 mM phosphate buffer of pH 7 containing 20% acetonitrile.

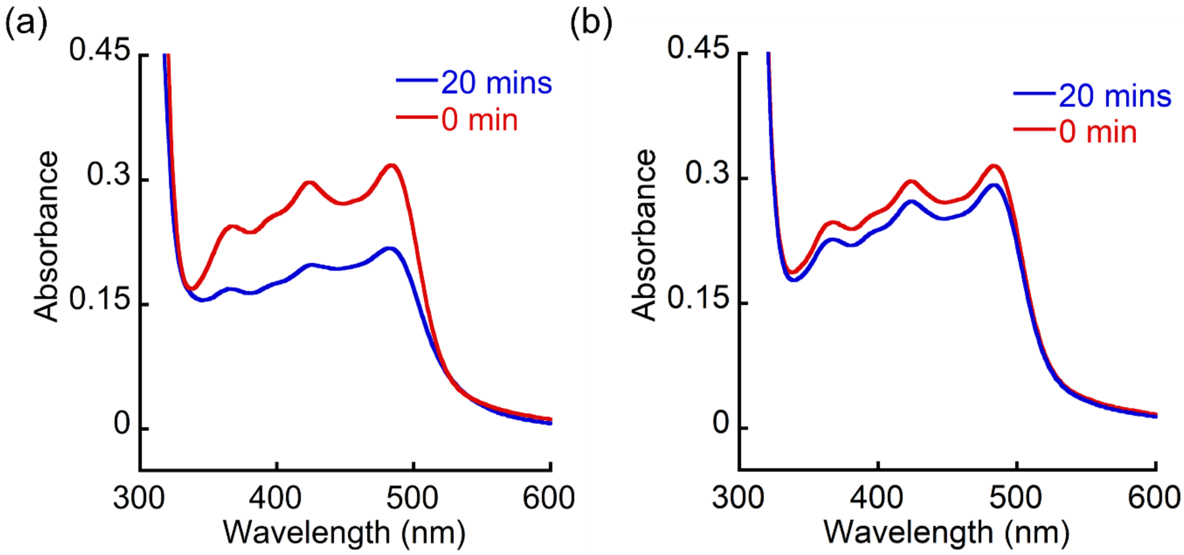

Figure S19. UV-Vis spectra recorded before and after 20 min of photolysis in 20 mM phosphate buffer (pH 7) containing 20% acetonitrile for the mixture of (A) PS2 and SEA or (B) Co**TBE**, PS2 and SEA (For the UV-Vis study 200 µL of the solution in 2 mL was taken from the mixture of 0.6 mM PS2 and 3.0 mM SEA or 25 µM Co**TBE**, 0.6 mM PS2 and 3.0 mM SEA).

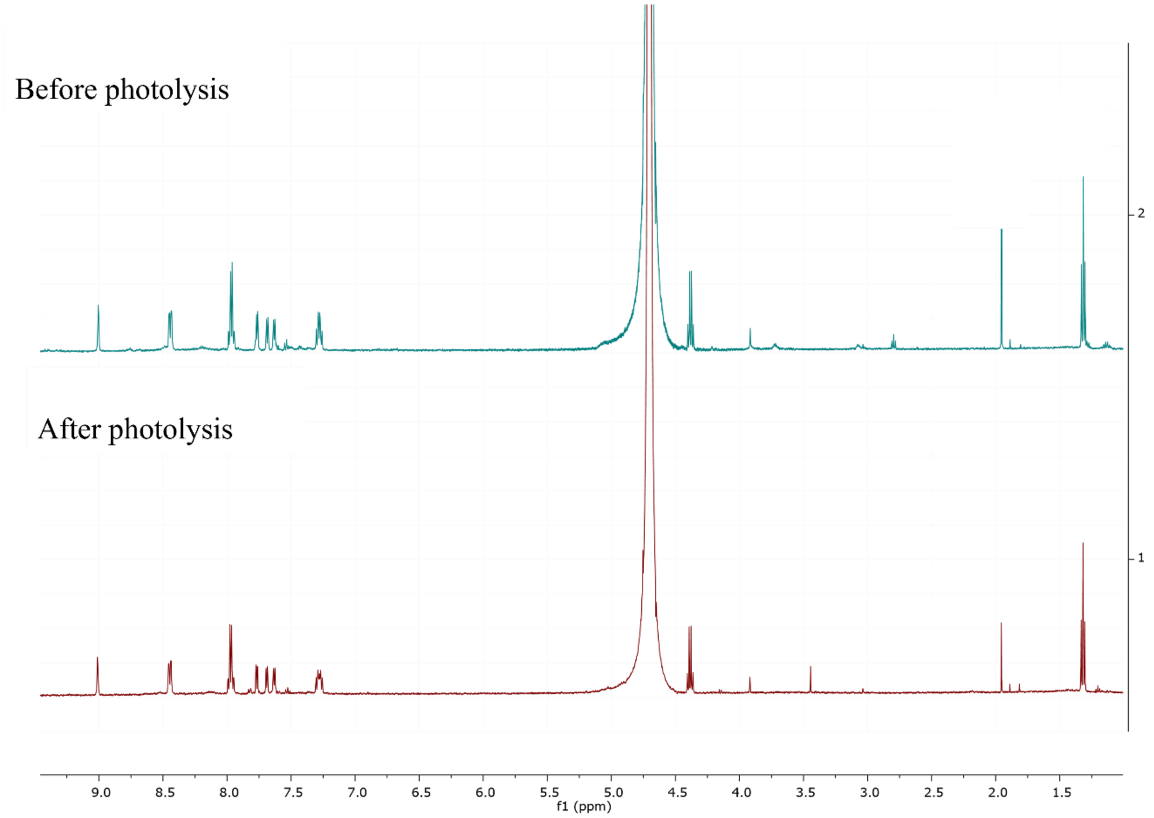

Figure S20. ^{1}H NMR in D_2O of the solution (20 mM phosphate buffer of pH 7 with 20% acetonitrile) containing 25 µM Co**TBE**, 0.6 mM PS2 and 3.0 mM SEA, recorded before and after 45 mins blue light irradiation.

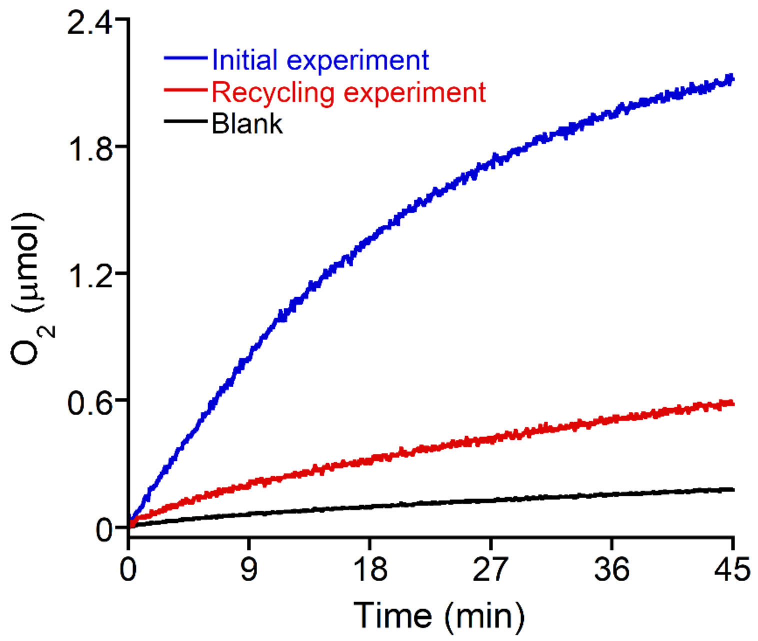

Figure S21. Photochemical oxygen evolution in the presence of 25 μM Co**TBE**, 0.6 mM PS2 and 3.0 mM SEA (blue curve). After the initial photolysis, fresh PS2 (0.6 mM) and SEA (3.0 mM) were added to the same reaction mixture, and the photolysis was repeated (red curve, recycling experiment) and the blank experiment with 0.6 mM PS2 and 3.0 mM SEA (black curve). All experiments were conducted under the irradiation of blue light in 20 mM phosphate buffer of pH 7 containing 20% acetonitrile.

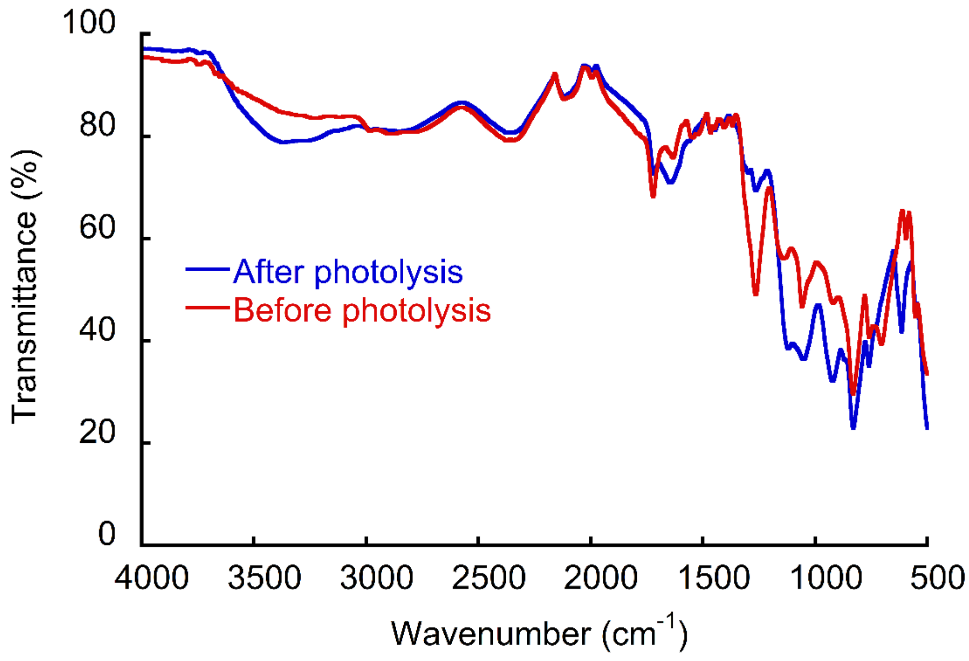

Figure S22. FTIR spectra of the mixture of solution containing 25 μM Co**TBE**, 0.6 mM PS2 and 3.0 mM SEA before (red) and after (blue) the photolysis for 45 mins in 20 mM phosphate buffer of pH 7 containing 20% acetonitrile (FTIR spectra were recorded as solids obtained by lyophilization of the liquid mixture).

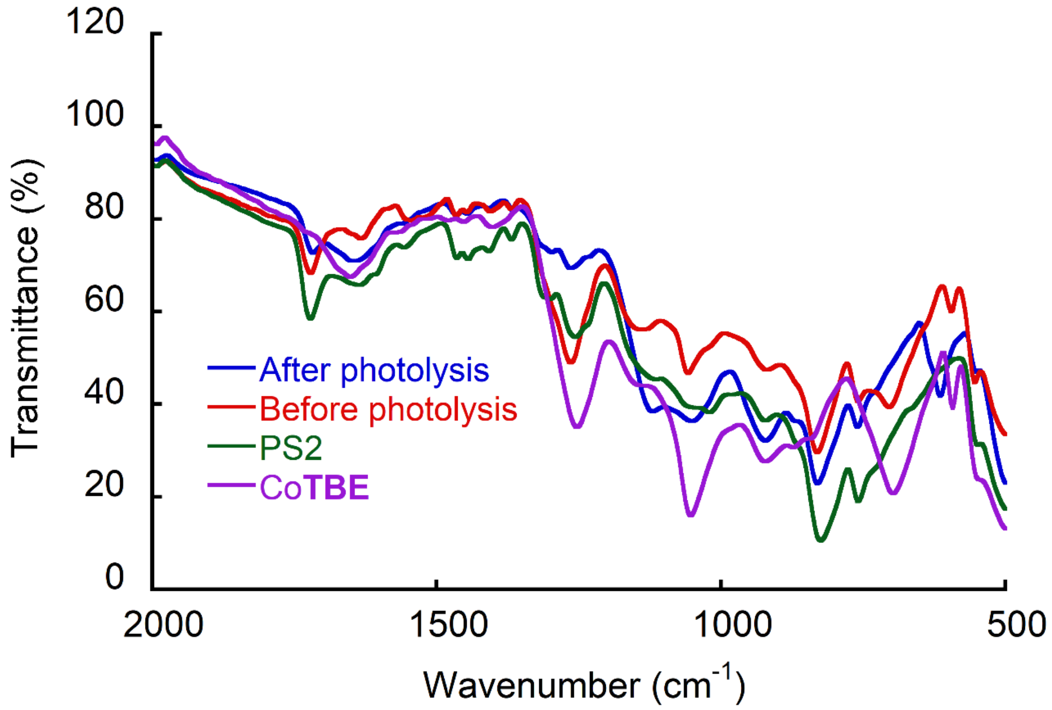

Figure S23. FTIR spectra of the mixture of solution containing 25 µM Co**TBE**, 0.6 mM PS2 and 3.0 mM SEA before (red) and after (blue) the photolysis for 45 mins, along with spectra of PS2 (green) and Co**TBE** (purple), in 20 mM phosphate buffer of pH 7 containing 20% acetonitrile (FTIR spectra were recorded as solids obtained by lyophilization of the liquid mixture).

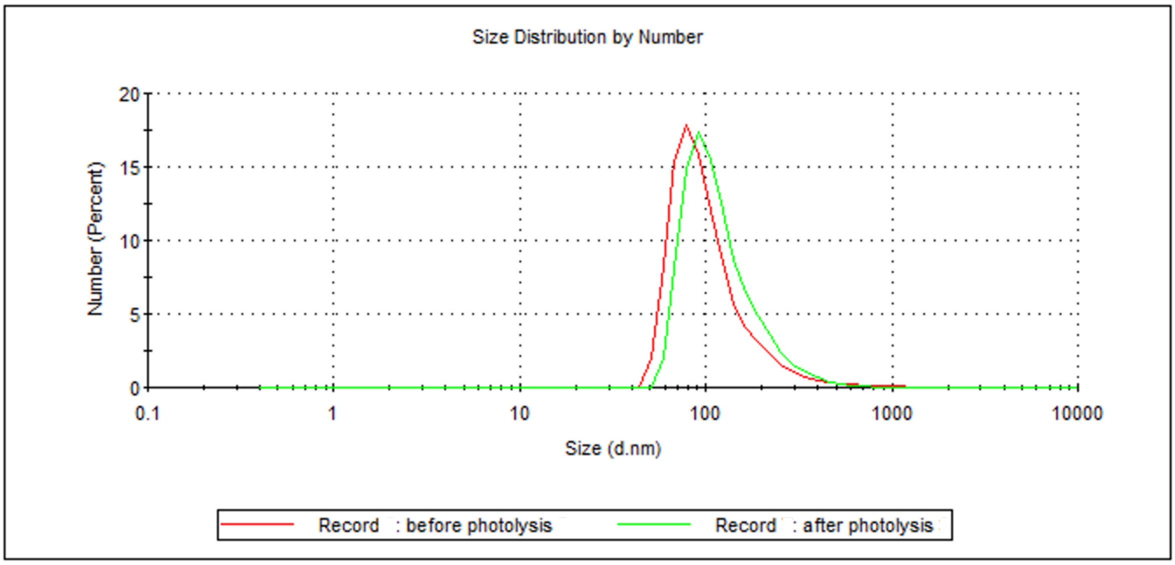

Figure S24. DLS spectra of the solution containing 25 µM Co**TBE**, 0.6 mM PS2 and 3.0 mM SEA before (red) and after (Green) the photolysis for 45 mins, in 20 mM phosphate buffer of pH 7 containing 20% acetonitrile.

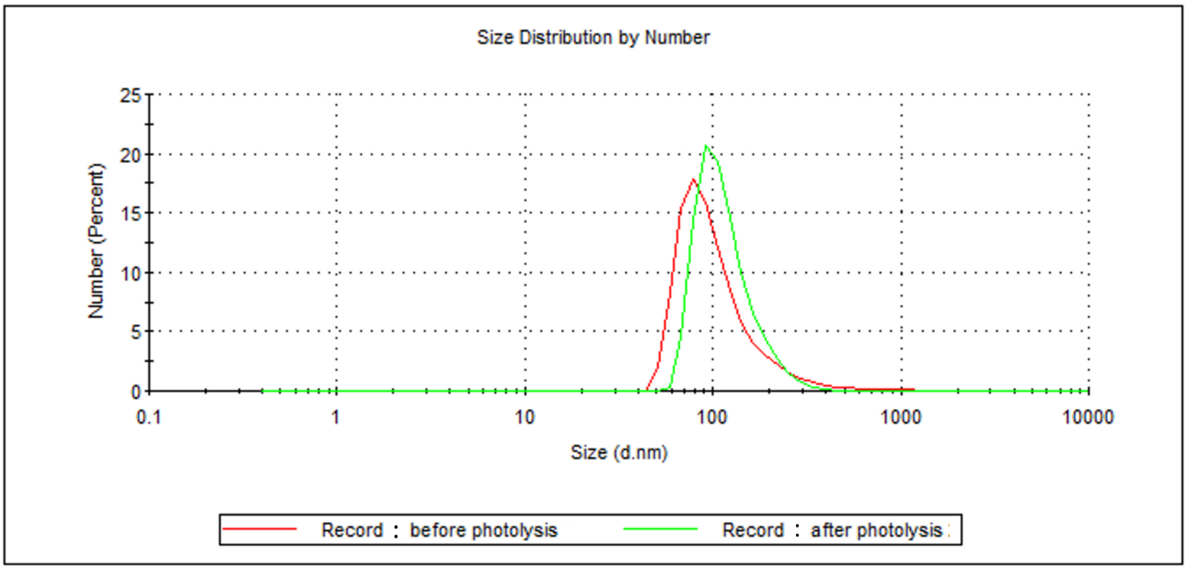

Figure S25. DLS spectra of the solution containing 0 μM Co**TBE**, 0.6 mM PS2 and 3.0 mM SEA before (red) and after (Green) the photolysis for 45 mins, in 20 mM phosphate buffer of pH 7 containing 20% acetonitrile.

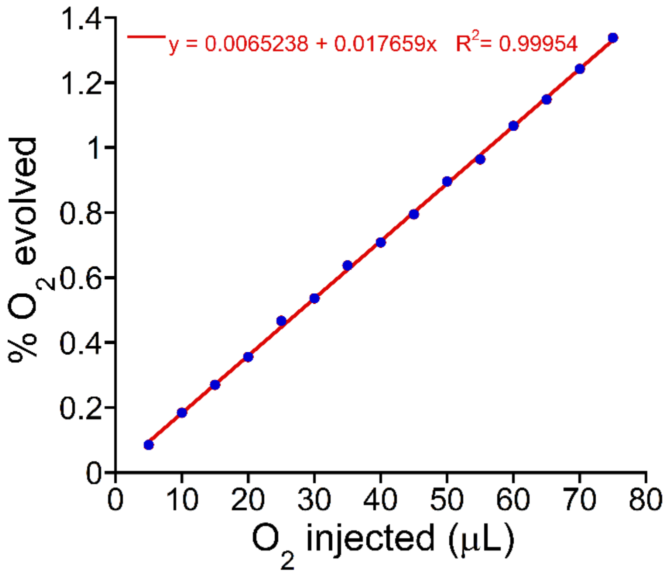

Figure S26. Calibration curve for the measure of evolved O2 in μL from $\%O_2$ (obtained from the oxygen sensor directly)**.**

Table S1. Initial optimization of the photocatalytic system in phosphate buffer (0.1 M) at pH 7.0 containing 20% of acetonitrile (the amount of oxygen evolved is reported after blank subtraction for each experiment at 45 min)

[Co**TBE**]/μM	[**PS2**]/mM	$[Na_2S_2O_8]$/mM	O_2/μmol
25μM	1 mM	5 mM	0.892 μmol
25μM	1 mM	4 mM	0.785 μmol
25μM	1 mM	3 mM	0.635 μmol
25μM	0.5 mM	2 mM	0.612 μmol
25μM	0.2 mM	1 mM	0.342 μmol
25μM	0.6 mM	3 mM	0.961 μmol
25μM	0.6 mM	5 mM	0.725 μmol

Table S2. Catalytic parameters of Co**TBE** in photochemical water oxidation experiment.

[Co**TBE**]/μM	Evolved O_2 (μmol) (in 45 min)	Evolved O_2 (μmol) (after blank subtraction in 45 min)	TON[b] (in 45 min)	TON[c] (in 5 min)	TOF[d] (s^{-1}) (initial 300 sec)	O_2 yield[e] (%) (in 45 min)
0	0.1712	---	---	---	---	5.70
5	0.6790	0.5078	50.78	11.42	0.038	22.63
10	1.051	0.8798	43.99	9.17	0.0305	35.03
15	1.2088	1.0376	34.58	10.36	0.0345	40.29
20	1.5266	1.3554	33.88	8.70	0.029	50.73
25	2.1044	1.9332	38.66	8.35	0.0278	70.13

[a]Conditions: Photocatalytic reaction with [Co**TBE**] = 5 μM (0.01 μmol) to 25 μM (0.05 μmol), [PS] = 0.6 mM, $[Na_2S_2O_8]$ = 3.0 mM in 20 mM phosphate buffer of pH 7 under the irradiation of blue LED. [b]TON (at 45 min) = μmoles of O_2/μmoles of catalyst. [c]TON (at 5 min) = μmoles of O_2/μmoles of catalyst. [d]TOF =$TON_{(initial\ 300\ s)}$ /300 s. [e]O_2 yield = moles of O_2/(1/2 × μmoles of $Na_2S_2O_8$). Moles of $Na_2S_2O_8$ used are 6×10^{-6} mol (6 μmol). TON, TOF and O_2 yield were calculated after blank substraction (values have been calculated according to **Figure S6**).

Table **S3**. Data summary of Co-based photocatalysts for homogeneous water oxidation in aqueous media from the reported literature

**Catalyst**	**Reaction Condition for catalyst, PS and SEA**	**Reaction medium**	**Light Intensity**	**TON**	**Reference**
Co**TBE**	5 μM catalyst, 0.6 mM PS2 and 3.0 mM $Na_2S_2O_8$	Phosphate buffer (20 mM, pH 7)	1.5 mW/cm^2 (blue LED)	51	This work
(TPA)Co(μ-OH)(μ-O_2)Co(TPA)]$(ClO_4)_3$	1.7 μM catalyst, 0.4 mM $[Ru(bpy)_3](ClO_4)_2$ and 3 mM $Na_2S_2O_8$	borate buffer (50 mM, pH 8).	LEDs (λ = 470±10 nm, 820 $\mu E/cm^2 \cdot s$)	58	S1
$[Co^{II}(Me_6tren)(OH_2)]^{2+}$	50 μM catalyst, 0.50 mM $[Ru(bpy)_3](ClO_4)_2$, 10 mM $Na_2S_2O_8$	phosphate buffer (50 mM, pH 8.0)	Xe lamp (500 W)	54	S2
$[Co^{III}(Cp^*)(bpy)(OH_2)]^{2+}$	50 μM catalyst, 0.50 mM $[Ru(bpy)_3](ClO_4)_2$, 10 mM $Na_2S_2O_8$	phosphate buffer (50 mM, pH 8.0)	Xe lamp (500 W)	29	S2
$[Co^{II}(qpy)(OH_2)_2](ClO_4)_2$	0.2 μM catalyst, 128 μM $[Ru(bpy)_3]Cl_2$, 10 mM $Na_2S_2O_8$	borate buffer (15 mM, pH 8.0)	500 W mercury arc lamp (457 nm)	335	S3
CoSlp	25 μM catalyst, 1 mM $[Ru(bpy)_3]Cl_2 \cdot 6H_2O$, and 5 mM $Na_2S_2O_8$	phosphate buffer (20 mM, pH = 7.1)	2.63 x 10^{-8} einstein/s (450 nm)	17.3	S4
$[Co^{II}(Py5OH)(Cl)](BF_4)$	10.0 μM catalyst, 0.15 mM	borate buffer (0.1 M, pH 8).	LEDs (λ = 470±10 nm,	51	S5

	$[Ru^{II}(bpy)_3](ClO_4)_2$ and 3 mM $Na_2S_2O_8$		820 μE/cm2·s).		
$[Co(Py)_2Slp]$	12.5 μM catalyst, 1.0 mM $[Ru^{IJ}(bpy)_3]Cl_2·6H_2O$ and 5.0 mM $Na_2S_2O_8$	phosphate buffer (0.1 M pH 9.0 or 20 mM, pH 7.0)	(500 W Xe lamp, λ ≥ 420 nm)	110	S6
$Co(TCA)(OH_2)_2$	100 μM catalyst, 1mM $[Ru(bpy)_3]Cl_2$ 10 mM $Na_2S_2O_8$	aqueous acetate buffer (0.1M, pH 7.5)	Xe-lamp (λ >420 nm)	90	S7
$[Co_2(\mu\text{-}OH)_2(TPA)_2](ClO_4)_4$	2.5 μM catalyst, $[Ru^{II}(bpy)_3](ClO_4)_2$ (3.1 nmol × 4) and $Na_2S_2O_8$ (0.25 mmol × 4)	borate buffer (50 mM, pH 9.3)	Xenon lamp (λ = 420 nm)	742	S8
$[Co^{III}(DPK·OH)_2]Cl$	0.2 μM catalyst, 1.0 mM $Ru(bpy)_3Cl_2$, 5.0 mM $Na_2S_2O_8$	sodium borate buffer (80 mM, pH 9.0);	LED lamp (λ 420 nm), 15.8mW	1610	S9
TPT_2Co	6.25 μM catalyst, 0.6 mM **PS1** and 3.0 mM $Na_2S_2O_8$	Phosphate buffer (20 mM, pH 9.5)	1.5 mW/cm^2 (blue LED)	28.1	S10
Co_2DNCH	1.50 μM catalyst, 0.10 mM $[Ru^{II}(bpy)_3](NO_3)_2$, and 4.0 mM $Na_2S_2O_8$	0.10 M borate buffer solution (pH 8.5)	300 W Xe lamp	1200	S11
$Co^{II}(Ch)$	catalyst (2.0 μM), $[Ru^{II}(bpy)_3](PF_6)_2$ (0.10 mM), and $Na_2S_2O_8$ (4 mM)	borate buffer (0.10 M, pH 9.0)	300 W Xe lamp	980	S12

References:

(S1) Wang, H.Y., Mijangos, E., Ott, S., Thapper, A., Water oxidation catalyzed by a dinuclear cobalt–polypyridine complex. *Angew. Chem. Int. Ed.* **2014**, *53*, 14499–14502

(S2) Hong, D., Jung, J., Park, J., Yamada, Y., Suenobu, T., Lee, Y.M., Nam, W., Fukuzumi, S., Water-soluble mononuclear cobalt complexes with organic ligands acting as precatalysts for efficient photocatalytic water oxidation. *Energy Environ. Sci.* **2012**, *5*, 7606–7616.

(S3) Leung, C.F., Ng, S.M., Ko, C.C., Man, W.L., Wu, J., Chen, L., Lau, T.C., A cobalt (II) quaterpyridine complex as a visible light-driven catalyst for both water oxidation and reduction. *Energy Environ. Sci.* **2012**, *5*, 7903–7907.

(S4) Pizzolato, E., Natali, M., Posocco, B., López, A.M., Bazzan, I., Di Valentin, M., Galloni, P., Conte, V., Bonchio, M., Scandola, F. and Sartorel, A., Light driven water oxidation by a single site cobalt salophen catalyst. Chem. Commun., **2013**, 49, 9941-9943.

(S5) Das, B., Orthaber, A., Ott, S., Thapper, A., Water oxidation catalysed by a mononuclear Co II polypyridine complex; possible reaction intermediates and the role of the chloride ligand. *Chem. Commun.* **2015**, *51*, 13074–13077.

(S6) Asraf, M.A., Younus, H.A., Ezugwu, C.I., Mehta, A. and Verpoort, F., Cobalt salophen complexes for light-driven water oxidation. Catal. Sci. Technol., **2016**, 6, 4271–4282.

(S7) Younus, H.A., Ahmad, N., Chughtai, A.H., Vandichel, M., Busch, M., Van Hecke, K., Yusubov, M., Song, S., Verpoort, F., A Robust Molecular Catalyst Generated In Situ for Photo- and Electrochemical Water Oxidation. *ChemSusChem* **2017**, *10*, 862–875.

(S8) Ishizuka T, Watanabe A, Kotani H, Hong D, Satonaka K, Wada T, Shiota Y, Yoshizawa K, Ohara K, Yamaguchi K, Kato S, Fukuzumi S, Kojima T. Homogeneous photocatalytic water oxidation with a dinuclear CoIII–pyridylmethylamine complex. Inorg. Chem. **2016**, 55, 1154–1164.

(S9) Zhao Y, Lin J, Liu Y, Ma B, Ding Y, Chen M. Efficient light-driven water oxidation catalyzed by a mononuclear cobalt (iii) complex. *Chem. Commun.,* **2015,** 51, 17309—17312

(S10) Pahar, S., Majee, K., Maayan, G., A Cobalt Complex from Terpyridine-Based Peptoid as an Efficient Catalyst for Visible Light Driven Water Oxidation, Eur. J. Inorg.Chem. **2024**, 27, e2023005,

(S11) Nakazono, T. and Wada, T., Photochemical Water Oxidation Using a Doubly N-Confused Hexaphyrin Dinuclear Cobalt Complex. *Inorg. Chem.* **2021**, 60, 1284–1288

(S12) Nakazono, T., Mitsuda, R., Hashimoto, K., Wada, T., Tamiaki, H. and Yamada, Y., The Catalytic Mechanism of a Highly Active Cobalt Chlorin Complex for Photocatalytic Water Oxidation. *Inorg. Chem.* **2024***,* 63, 24041–24048
